# “…and How Are the Kids?” Psychoeducation for Adult Patients With Depressive and/or Anxiety Disorders: A Pilot Study

**DOI:** 10.3389/fpsyt.2019.00004

**Published:** 2019-02-05

**Authors:** Marieke R. Potijk, Louisa M. Drost, Petra J. Havinga, Catharina A. Hartman, Robert A. Schoevers

**Affiliations:** University Center for Psychiatry, University Medical Center Groningen, Groningen, Netherlands

**Keywords:** anxiety, depression, implementation, parental support, child wellbeing, prevention

## Abstract

Depressive and anxiety disorders are highly prevalent and form a substantial burden for individuals and their family members. A recent study showed that approximately two-thirds of the children of patients with severe depressive and/or anxiety disorders develop one of these disorders themselves before 35 years of age. In the Netherlands, various preventive interventions are available for children of parents with mental illnesses. However, the actual reach of interventions is small (< 1% of all children participate). A major barrier appeared to be parents' hesitancy to let children participate in preventive programs. In order to address this barrier, we designed a pilot study to implement a psychoeducation program on parenting and mental illnesses. The main aim of this study was to investigate how a preventive family-focused approach can be embedded in routine adult psychiatric care. The pilot started in April 2017 and has ended in September 2018. It was conducted in the University Center Psychiatry (UCP) in Groningen, The Netherlands. This article describes the implementation process so far. The main intervention was a monthly organized group-psychoeducation called “parenting and a mental illness,” which could be attended by parents currently treated in the UCP. In 18 months, implementation activities were divided in four phases; (1) Creating awareness, (2) Adoption of the intervention, (3) Implementation and evaluation, and (4) Continuation phase. The program development process was evaluated using both qualitative and quantitative data. Based on the pilot study we were able to make recommendations for the further implementation of this approach so that more parents can be reached in future. Further study with follow-up activities is needed to measure the effects of the psychoeducation, for instance on parenting functioning and the quality of the parent-child interaction.

## Introduction

Up to 45% of patients who receive adult psychiatric care are parents (1). Having a parent with a mental illness greatly affects children's wellbeing. Approximately two-thirds of the children of patients with severe depressive and/or anxiety disorders develop such a disorder themselves before their 35th year, and half of them even before their 20th year (2). These children form an important target group for prevention (3).

In the Netherlands, various interventions for prevention of mental health problems in offspring of depressive and anxious adults have been developed. Current interventions are aiming at the parents (psycho-education and parenting skills), the children (psycho-education, coping skills, mutual support), or the family as a whole (group sessions, online support) (4, 5).

However, the vast majority of children and parents does not receive the services they require (6). Regarding the Dutch preventive interventions aiming at the offspring of parents with a mental illness, no more than 1% of children take part (4, 7). The primary challenge, therefore, is to increase the reach of the preventive interventions for parents and children.

A first and crucial step in increasing the range of participants in preventive interventions is to get the offspring in the picture. A substantial group of these high-risk children can easily be detected; namely through the parent being treated. van Doesum et al. (8) found that mental health agencies are the most common source of referrals to preventive programs for offspring of parents with a mental illness. Moreover, screening on the presence of children in adult psychiatric care has already been improved in several countries. In Norway health legislation was altered in 2010, making it mandatory to assess whether or not psychiatric patients have children (9). In the Netherlands, it has been obliged to register the number of minor children of adult psychiatric patients, since 2017.

However, Skogøy et al. (10) underline that legal changes are helpful, but not sufficient to secure equal chances of protection and support for children of parents with mental illnesses. Research has indicated that both parents and professionals experience several barriers to be involved in prevention. Barriers of parents include: perceived lack of necessity for intervention, overburden, reluctance to bother their offspring, concern for shame and stigma, and practical problems such as lack of transport facilities (8, 11–13)

Professionals may perceive talking about parenthood with their patients as a sensitive topic (8, 14–16). Furthermore, between disciplines differences may exist in the ease with which professionals talk about parenting and wellbeing of the children. Findings of Maybery et al. (17) suggest that social workers feel more comfortable with talking about these family issues than nurses working in adult psychiatric care. Foster et al. (18) however, argue that nurses in mental health care are in prime positions to support parents who are patients. These authors proposed a framework for family-focused actions that psychiatric nurses can carry out at different phases of client care, such as providing age-appropriate information on mental illness to children and facilitating parental attachment with offspring. Furthermore, based on consultation with a group of senior clinical leaders in adult mental health services in Australia, Goodyear et al. (19) introduced essential and explicit practice standards for mental health nurses working with families. These efforts illustrate a growing knowledge base of what should be implemented into adult health care to serve patients who are parents and their children. However, papers on program development that investigate how knowledge can be successfully translated into clinical practice are scarce.

## Aim of the Current Study

The main aim of this pilot study was to investigate how a preventive family-focused approach can be embedded in routine adult psychiatric care. This aim resulted in the following research questions:

1. How can a preventive family-focused approach be made feasible and acceptable, for parents and professionals?
2. Which remedies can be identified in order to increase feasibility and acceptability?
3. Which recommendations can be given to further implement this approach?

## Methods

### Design

We designed a pilot study to implement a preventive, family-focused approach for adult patients treated for depressive and/or anxiety disorders. We tried to build on existing routines so that changes were kept as small as possible. In the University Center Psychiatry (UCP) a psychoeducation program for parents already existed for a few years, but the program was not offered on a structured base, i.e., at maximum three to four times a year, at different times and dependent on the number of parents staying in the inpatient ward. A social worker of the inpatient ward coordinated these sessions and any other actions for patients regarding their children. Also, she led a special working group of professionals. Nurses were used to refer to this social worker, who invited parents to talk about the impact of parents' illness and to help explain the situation to children, on an age appropriate level.

In a four-phase implementation plan, we worked toward a situation in which all professionals mention patient's role as a parent at least once during routine treatment, and motivate parents to attend the group-psychoeducation “parenting and a mental illness” which was now offered monthly. Since it is known that parents may perceive different kinds of barriers to participate in preventive interventions, we listed common barriers and made a plan to address them in this pilot study (Table 1).

**Table 1:** Overview of the implementation process.

**Barriers**	**Initial approach**	**Feasibility**	**Acceptability**	**Adjustment**	**Recommendations**
Perceived lack of necessity to talk about parenthood and mental health	Creating awareness by providing information about the project e.g., flyers, newsletter, survey of professionals' attitudes	Overload of professionals	Professionals' view that talking about parenthood is important but does not belong to their core tasks Patients do not mention the subject spontaneously when the professional skips it	Involve management to make talking about parenthood part of the department policy Informative, normalizing online video (infographic) to increase awareness and acceptability Website with possibility for parents to sign up for a psycho-educative session themselves	Consideration of appointing an attention officer for patients' offspring Involvement of management needed to ensure that every patient who is a parent receives information about mental health and parenting Improvement of ICT facilities in electronic patient files, for e.g., a button “parenthood” and a treatment plan on parenting and child wellbeing
Reluctance to bother offspring; unfamiliarity with possibilities for prevention	Professionals should make the parent understand the importance of information for children.	Parents need support how to talk about their illness with their children	Parents' anxiety to loose custody	Relation building with parents, empathy for being reluctant while emphasizing the importance of information Peer exchange of parents' experiences; Information about further referral possibilities	Collaboration with other disciplines (public health, gp's) to continue and improve support for families
Feelings of shame and guilt (“being a bad parent”)	Normalization of the attention for patients' children and their parenting roles	It appeared difficult for professionals to apply a family focused approach (which is broader than child safety procedures)	Different opinions among professionals regarding their task; who can best deal with family matters of patients and when?	Project leader attends the team meetings of all participating wards to discuss how this “child-aware” approach could best be implemented into work processes in their ward	Training of professionals how to introduce and talk about parenting with patients
Overload of the parent	Make professionals mention the topic at least once during therapy Refer to existing 2-h session of psycho-education	Acute phases of decompensation may ask for alternative ways and/or moments to raise the topic Group sessions can only take place with sufficient participation	Patients' feedback: timing is important when to talk about parenting	If there are reservations regarding the timing of mentioning parenthood, the timing will be discussed in the team meeting Psycho-education sessions are offered on a structural base to improve feasibility	Create guidelines how and when to talk about parenting and when to refer parents to the psychoeducation meeting Discussing parenthood should be adjusted to patient's condition (but will not be skipped) Automatic notifications in electronic patient file to reconsider action of practitioners at a later moment
It appeared difficult for professionals not to focus mainly on child safety procedures	Training for professionals	Due to an overcrowded training schedule, the training is postponed	Professionals differed in opinion about who could best deal with family matters of patients	Project leader attends the team meetings of all participating wards to discuss how this “child-aware” approach could best be implemented into the work processes in their ward	Postponed training will be scheduled Recommendation to document the working procedures for this point in each department

The medical ethical committee of the University Medical Center Groningen evaluated this study as not falling under the Medical Research Involving Human Subjects Act (WMO). Nevertheless, we followed the required procedures and obtained written informed consent for the qualitative components of the project. No written informed consent was requested for participation in a psychoeducation meeting because this option was offered as part of the treatment. Patients knew that their own therapist would be informed of their participation. Collection of participant information was kept to a minimum and reported at group level, such as the number of participants who joined the psychoeducation. In (online) registration the current privacy legislation was followed and only the necessary information was requested, such as an e-mail address confirming the registration, and the age category of the children to adjust the psycho-education on that particular evening.

### Participants

The target group consisted of adult patients who are parents of children in the age range of zero till 24 and who are currently being treated for depressive and/or anxiety disorders in an outpatient or inpatient ward. Patients will be informed by their own practitioner (psychologist, psychiatrist, psychology/psychiatry resident, or nurse practitioner), social workers, and/or nurses of the inpatient wards. The participating wards are one outpatient ward and two inpatient wards for patients with depressive disorders, 2 day-clinics for depressive disorders, one outpatient ward for bipolar disorders, an outpatient ward for anxiety disorders, and a day-clinic for anxiety disorders. Since the UCP is a tertiary care center, symptom severity of most patients is moderate to very severe.

In 1 year about 1,200 adult patients with depressive and/or anxiety disorders are treated in the UCP. It has been estimated that 30–50% of these patients have children. Systematically, information on the parent status of a patient is gathered when patients enter the UCP to receive care, via a general questionnaire. In this way practitioners know by forehand (a) whether the new patient is a parent, (b) how many children he or she has, and (c) what the age of the children is.

### Primary Intervention: A Preventive, Family-Focused Approach

We performed this pilot to investigate the possibilities to permanently embed a preventive, family-focused approach in routine treatment protocols. In concrete terms, this meant that:

1. Practitioners and/or nurses mention patient's role as a parent at least once during routine treatment, and motivate parents to attend the group-psychoeducation “parenting and a mental illness.”
2. Psychoeducation meetings for patient-parents are offered on a regular base.
3. We examined possibilities to permanently embed the preventive, family-focused approach in the organization and management structures.

### Psychoeducation “Parenting and a Mental Illness”

We organized a monthly group-psychoeducation, on every first Thursday evening of the month, called “parenting and a mental illness.” An experienced social worker of the inpatient ward for depressive disorders of the UCP and a child psychologist gave the psychoeducation, using a power point presentation. Project leader MP (resident psychiatry) coordinated applications for the evenings and was available to stand in when one of the group leaders was hindered to give the psychoeducation. Patients attended one meeting and were encouraged to bring their partners. The psychoeducation could only be attended by parents currently treated in the UCP. The content of the psychoeducation was theory-based and consisted of the following main items:

- Patients introduce themselves shortly (name, family situation, the current treatment status), followed by an introduction to the theme parenting and a mental illness. In particular, attention is paid to illness perception and the relation with the patient's role as a parent, before and during treatment. Group leaders pay attention to feelings of guilt or shame that may be present regarding shortcomings in the parenting role. Furthermore, the role of the other parent is a topic of conversation.
- How do parents tell their children about mental illness? This starts as an open conversation in which parents share their experiences. Later, group leaders give a summary and provide examples of how to talk with children about their mental illness, using age-appropriate information. Group leaders may notice barriers in talking with their children among parents, for example not wanting to bother their children and bring up these subjects.
- How do parents know when it is (not) going well with their children? Do they have worries about their children's wellbeing? For each age category, the group leaders provide information on signals of (ab)normal development. Sharing of experiences among parents is encouraged.

Additionally, at the end of the psychoeducation meeting further supportive options were offered to the parents, such as continued conversations about parenting with their current practitioner, or other professionals (e.g., the general practitioner, a social worker). If necessary, referral of the child for child psychiatric consultation was offered.

We estimated that 68 parents would have attended the group-psychoeducation after 1 year, based on data that about 1,200 patients with a depressive and/or anxiety disorder are treated in the UCP in a year. About 480 of the patients have children (40%) and we estimated that 336 parents could be motivated by practitioners to join the psychoeducation, with an eventual reach of 20% of the motivated parents.

### Implementation Procedure

The pilot study started in April 2017 and was planned to end at the end of September 2018. In this time span of 18 months, implementation activities were divided in four phases; (1) Creating awareness, (2) Adoption of the intervention, (3) Implementation and evaluation (following an iterative process), and (4) Continuation phase. We described intended activities in these four phases in our implementation plan that we wrote prior to the start of the project. The implementation plan, including the four phases, was based on a workbook for guideline implementation in youth help and protection services (20).

#### Phase 1: Creating Awareness Among Parents and Professionals (April–October 2017)

During the first months of the project, we spread information about the project via various ways. In April we announced the project in the general newsletter of the UCP. During the next months, the project leader (MP) attended the regular team meetings of all participating wards, to discuss with colleagues how the family-focused approach could be embedded in their ward. In addition, these meetings served to spread flyers with practical information about the project and to present a flow chart of general procedures regarding children of patients and child safety. At the launch of the actual implementation (September 2017), more information on the project was given in a presentation for all professionals and researchers in the UCP. We stressed that the primary aim of the project was to pay more attention to patients' normal role as a parent, for example by asking how parents manage to perform this role now they are being treated for a mental illness. Existing child safety procedures were mentioned, but were not the main focus of the project.

Finally, we created awareness for the importance of implementing a preventive, family-focused approach. For both parents and professionals, we developed an infographic film of 3 min illustrating the impact on the family when one of the parents has a mental illness. This infographic was placed on the project website https://kopp.umcg.nl that we had developed in this first phase of the implementation. The launch of the project (including the infographic) was also spread as a local news item by the press agency of the UMCG, among others via Facebook. On the project website, more information was provided regarding the monthly group-psychoeducation on parenting and a mental illness. Recently, the infographic was translated to an English version which can be found via the following link https://www.youtube.com/watch?v=mZbsXjh2hYM.

#### Phase 2: Adoption of the Intervention (September–December 2017)

This phase predominantly focused on activities that promoted the adoption of the intervention, such as creating awareness and support of various groups, including the management.

First, via a survey among professionals of the department mood and anxiety disorders, we inventoried general practices regarding the attention for patients' role as a parent in routine treatment protocols. This raised awareness among participants and gave us an impression of the current state of preventive, family-focused care at the department. Second, in order to gain broad support for the project, we organized participation meetings with the client counsel, social workers, residents, the secretaries, and managers of the department. About once in 3 months, we wrote an update of the implementation process to publish in the UCP newsletter.

#### Phase 3: Implementation and Evaluation (September 2017–August 2018)

From September 2017 on it was possible to sign up for the group-psychoeducation sessions. Both parents themselves and practitioners were able to sign up, online via the project website, via an email or via a registration form on paper. The first psychoeducation session took place on the first Thursday in October 2017. After the start of the psychoeducation sessions, we constantly adjusted the original implementation plan to the actual situation in clinical practice, using an iterative approach. Feedback of parents and practitioners was essential during this phase. For example, after introducing the pilot project, the importance of the topic “parenting and a mental illness” was endorsed by many professionals. However, some of them indicated that they had not enough time to discuss parenting in the treatment sessions within the scheduled time. To solve this problem, we suggested that they could use the infographic film of 3 min to introduce the topic and refer parents to the group-psychoeducation. Another example of a change in the initial plan was prompted by the observed difference in working methods at the various wards. By attending team meetings of the wards, we explored together with the professionals how the family-focused approach could best fit the current working methods in their opinion. Furthermore, parents were asked to fill out a feedback form after attending a meeting (Supplement 1) and some of them were asked for a telephone interview. This information was used to help to define barriers and remedies (Table 1).

Parallel to the implementation of the family-focused approach, the experiences of nurses with talking about parenting were investigated by students of the School of Nursing (University of Applied sciences). They performed two qualitative research projects, in which nurses were interviewed both individually and in focus groups (which included other professionals). For the interviews, topic lists on the theme parenting were used. With respect to the aim of this study, we do not extensively report on the nursing projects in this article. However, these projects may have contributed to increased awareness of parenting and continued positive reinforcement. In general, nurses were highly motivated to participate in the interviews and brought up some interesting topics, such as task ambivalence. On the one hand they felt family-focused care as one of their core responsibilities, but on the other hand, many nurses found it unclear when and how specific tasks should be performed and by which professional.

#### Phase 4: Continuation (September–December 2018)

The main goals of this final phase of the implementation were (a) to create support and guidance for the intervention in the organization and management structures, (b) to realize professional guidance on the work floor and/or training, and (c) to make arrangements with management and stakeholders for continuation of the project. To date, we have taken the following steps. First, we have developed the parental questionnaire “parenting and a mental illness,” involving questions on experiences of patients' role as a parent and wellbeing of the children (Supplement 2). The reason for developing this questionnaire was to ease conversations about parenting between parents and practitioners or nurses. It is now available for use as part of the routine outcome measurements (ROM) of the department. Patients who have started a treatment automatically receive an email with an invitation to fill out the parental questionnaire (online). When the questionnaire is completed, the practitioner of the patient can see the answers in the electronic patient file. In this way, we expect that practitioners will have a better view of the family situation, e.g., how patients perceive their parenting role, which may ease family-focused conversations with the patient. Evaluation of the use and effectiveness of the questionnaire will take place at a later stage. Second, to meet the wish of professionals who indicated they needed more practical support, we set up a targeted training. However, eventually, we decided not to schedule the training since it was not considered realistic in a year in which two new electronic patient registration systems were introduced. Therefore, training programs have been designated as a future priority. Lastly, we asked the management and stakeholders to express the intention to continue the family-focused approach as part of routine treatment protocols. This has already resulted in initiatives such as including information on mental illness and parenthood in the training program of psychiatry residents.

### Analysis

The implementation was evaluated using both quantitative and qualitative data. The quantitative part consisted of a survey among professionals of the department of mood and anxiety disorders and registration of the number of participants at the monthly group-psychoeducation meetings. The online survey (Supplement 3) was created using the program Survey Monkey and was accessible for the professionals via an email that was sent by the secretaries. We asked ten multiple choice questions which were determined through discussions in the project group (MP, LD, PH, CH, RS). MP analyzed the outcomes of the survey, which are documented in a word file (anonymous). Registration of the actual number of participants was done by social worker HH, who was present at all but one psychoeducation meetings, and further processed in an SPSS file by MP. All data were checked and verified by at least two researchers (mostly by MP and LD).

The qualitative part consisted of written evaluation forms of the psychoeducation meetings (filled out by parents) and semi-structured telephone interviews with some of the parents who participated in a psychoeducation meeting. Questions asked were for example: “What was the most positive of the evening and what are you least satisfied with?” and “Do you have any suggestions for us to improve this psycho-education?”

Lastly, we reflected on the barriers that we had listed, whether and how we addressed them and which remedies were found (Table 1).

## Results

### Survey

In September 2017, at the launch of the implementation, we performed a survey among professionals of the department of mood and anxiety disorders. All professionals received an email with a link to the online survey which consisted of ten multiple choice questions about the general way of practice regarding family-focused care. Thirty-two professionals filled out the survey in a time span of 6 weeks. Most of them were nurses (13/32), followed by psychologists (7/32) and other professionals, such as creative therapists (5/21). Furthermore, three psychiatrists and four residents in psychiatry filled out the survey. Respondents were about equally spread among the different wards of the department of mood and anxiety disorders; professionals from the inpatient wards (12), the day-clinic wards (11), and the outpatient wards (13) were represented. Some professionals worked at more than one ward. Nineteen of the 32 respondents (59.4%) answered that they mostly or always mention parenthood when they talk to patients who have children in the age of 0–24 years. The other 13 respondents answered they do not mention the topic regularly. Regarding the timing for bringing up the topic, most respondents thought the beginning of treatment (until the third meeting with the parent) is most suitable. Although most professionals said to talk about parenting and/or child wellbeing, they indicate a need for further education or skills training in their current work. Eleven professionals want to have more general information on mental illness and parenthood; for example on the actual risk that children will develop problems if their parents have a mental illness. Furthermore, 19 professionals indicate that they miss practical information, such as to which organization they can refer parents and/or children if they signal any problems. Finally, skills training in conducting conversations about parenting and child wellbeing is wanted, as indicated by 11 professionals.

### Psychoeducation “Parenting and a Mental Illness”

In Table 2 we show the number of parents that participated in the psychoeducation meetings. The number of participants varied per meeting. In the meeting of June, the highest number of parents participated, i.e., 12 parents (Table 2). In July only one person applied, probably that was due to the summer holidays. Most patients came with their partner or, in exception, with another family member. Twelve patients came alone. After December 2017, when we had launched the online possibility for registration, the majority of the participants applied via this way. In total 64 parents participated in the psychoeducation meetings, which was lower than the estimated number of 68 at the start of the study. Regarding the age of the children that were reached via psychoeducation, we are only able to provide a rough estimate in age groups. Most parents that applied for a psychoeducation meeting had one or more children in the age ranges of 4–11 (at least 18 children) and 12–17 (at least 14 children). Fewer parents had one or more children in the age ranges of 0–3 (at least 9 children) and 18+ (at least 4 children). For 6 participants data were missing regarding the age of the children.

**Table 2:** Number of parents^*^ participating in the psychoeducation meetings.

**Date**	**Number of participants**	**Way of application**			
	**(Actual/ applied)**	**Via practitioner**	**Written form**	**Online form**	**e-mail or other**
2017/10/05	8/10	2	2	NA	4
2017/11/17	4/4	.	.	NA	3
2017/12/17	4/6	0	0	4	2
2018/01/04	2/3^#^	0	0	3	0
2018/02/01	4/6	0	4	2	0
2018/03/01	8/8	.	.	5	1
2018/04/05	6/6	.	.	5	.
2018/05/03	4/4	0	0	4	0
2018/06/07	12/12	4	0	8	0
2018/07/05	1/1^#^	0	0	1	0
2018/08/02	9/9	4	1	4	0
2018/09/06	2/4	0	0	4	0
**Total**	**64/73**	**10**	**7**	**38**	**10**

* *in most cases the other parent came along with the patient; in a few cases a grant parent or other family member was present*.

# *individual appointment via social worker instead of the regular group psychoeducation*.

At the end of the psychoeducation meetings, we asked participants to fill out an evaluation form in which we asked whether parents regarded the meeting as useful and which needs they had regarding parenting and wellbeing of the children. Twenty-five participants filled out the evaluation form. All regarded the meeting as useful. Most of them further commented on this question, as shown in Table 3. Of the 25 participants, 12 regarded the information of the meeting as sufficient, 2 expressed the need for further information, 3 preferred to talk about parenting more frequently with a nurse of the ward, 6 would like to go to a social worker or therapist, and 2 had other preferences for further support. One patient who preferred to talk about parenting with a nurse said: “It keeps me bothering whether the situation at home is good, whether it is safe enough, comfortable, and also a bit “normal.” A parent expressing other preferences wished for: “The possibility for our children to talk with someone.”

**Table 3:** Reactions of parents who attended a psychoeducation meeting.

**Question: what did you find useful of this meeting?**
“It was nice to share our experiences with other parents”
“It was a relief to know that many things are going well in the way we approach the children regarding my illness”
“It gave me insights in the possible impact of my illness on the children, and that early signaling is important to prevent that they will have problems”
“The importance to keep in touch with our children”
“Some things I recognized, other things are not applicable to our family”
“It was confronting (we recognized the impact of my illness on our son), and we now have more knowledge than before this meeting”
“Now we know that we need to talk about it with the children”

In addition, we interviewed four participants by telephone after they had attended a group-psychoeducation session. The participants who were interviewed concerned one woman who had been treated for depression and her husband and two women who were partners of patients in care. Beforehand they all consented to be interviewed about their experiences. All four were positive about the organization and contents of the evening. Exchange of experiences was mostly appreciated, although sometimes confrontational, for example when it appeared that other participants had received more familial support than the respondent. The timing (to offer this intervention during hospitalization) was seen as very important. Recommendations on which words to use to explain about the mental illness to their children were appreciated. One of the respondents suggested that this meeting should be mandatory for all patients with children.

### Barriers and Remedies

Table 1 shows how the implementation process changed during the course of the project. The initial implementation plan was based on the barriers for parents to talk about their parenting we knew from the literature. With the help of the logbook, it was recorded which parts of the plan were feasible and acceptable and which were not (found barriers). Solutions were sought and found in consultation with those involved. Successful adjustments to the implementation procedure (remedies) were, for instance, the introduction of the online infographic, the discussions in the team meetings, involving the management, and the website with possibility for parents to sign in for a psycho-education session themselves. At the end of the project an overview could be made of the recommendations we deemed necessary for a successful continuation of the implementation, such as improvement of ICT facilities and the scheduling of the training that was postponed.

## Discussion

In this pilot study, we have described the development of a program to implement a preventive family-focused approach into routine care for adult patients who are parents and treated for moderate to severe depressive and/or anxiety disorders. The focus was on feasibility and acceptability. Most professionals expressed their sympathy to the project. Our survey showed that professionals consider it as important to inform patients who are parents about the possible impact of mental illness on parenting (nearly 60% of professionals already talked about parenthood and child wellbeing with their patients at the start of the implementation). The other 40% did not mention the topic frequently, and the need for additional training (education and/or skills) was indicated by a majority of professionals.

Team discussions revealed that opinions differed between disciplines whether talking about parenthood was part of their job. At wards where a social worker was part of the team, patients were preferably referred to the social worker for further consultation. However, the option of referral to a social worker is not present at all wards of the UCP. It gradually became clear that the family-focused approach fitted very well with the core business of nurses. First of all, the survey was filled out by nurses for 41% (13/32). This fits with literature on this topic, which shows that nurses are in a unique position to initiate conversations on parenthood and child wellbeing because of their direct, frequent, and sustained contact with patients and families (18). Nevertheless, family-focused care should not be reserved for specialized professionals. Small talk about normal life (events), including parenting, can, for example, be initiated by a secretary who can also hand over a leaflet with information about targeted psychoeducation. Furthermore, therapists of all kinds can stress that mental illness may have impact on parenting and that they, therefore, want to pay attention to it during therapy sessions. Management support and professional education in a positive organizational culture (13) are conditions to realize a therapeutic climate where support of patients who are parents becomes a normal part of treatment. This can benefit both parents and children (21).

As Foster and Isobel (14) have pointed out, talking about parenting involves special capacities. In our survey, 11 out of 32 professionals expressed their interest in training opportunities to address parenting and child wellbeing in conversations with patients. Other respondents mentioned (and perhaps rightly so) that they had enough conversation techniques to be capable to discuss parenthood. Interviews and case discussions during the team meetings provided more insight into the emerging dilemmas of professionals, such as the idea of having to choose between the good of the parent and that of the child (22). Reupert et al. (13) warn that professionals, including social workers and nurses, seem to avoid such dilemmas by arguing that talking about parenthood can harm the confidentiality of the relation between professional and patient. They also hold a plea to be creative in finding a solution when there is the conviction that these conversations are necessary.

The monthly organized psychoeducation meetings for parents were visited by 64 parents in 1 year. By far most of them (38) applied via the online registration form on the project website. Parents' reactions after the psychoeducational meetings were mainly positive, and they provided useful feedback to further improve the meetings. A good timing of the psychoeducation, for instance, was deemed indispensable. Parents' reactions were discussed in the team meetings by the project leader and once more colleagues were asked to draw their patients' attention (on time) to the educational evenings. This illustrates that the implementation of a preventive approach is not achieved after one or two actions, but requires continuous positive reinforcement (23).

The number of parents participating in the monthly psychoeducation meetings was variable. More professionals started to refer parents, but the most visible increase in registrations was in the number of parents who applied directly via the website. This may indicate that the psychoeducation meets the needs of, at least a subset of parents treated in the UCP, which also corresponds with the positive feedback of parents who had joined a psychoeducation meeting: all regarded the meeting as useful, and about half of the parents would like further support. Furthermore, feedback and interviews indicated that awareness among parents about the impact of the mental illness on child wellbeing has grown. We think that for e.g., better provision of information about the preventive offer may have facilitated parents to be more aware and to participate in the psychoeducation. Furthermore, the easy way of registration may also explain the higher number of online applications, or the fact that the involvement of a (busy) professional can be avoided.

A limitation of our research is that it was carried out in a specific setting under specific circumstances. It is unlikely that the same circumstances are present in any other organization for adult care, which could result in different outcomes. On the other hand, a strength of this pilot is that we were able to demonstrate how an iterative implementation method can be used to realize a translation from current knowledge to clinical practice. Further prospective studies with a control group are needed to assess whether preventive psychoeducation, as part of a family-focused approach, is effective. Findings of this pilot study provide useful information for setting up larger studies with a higher impact. Our aim is to continue the psychoeducation program in the UCP and to further develop it, using the outcomes of this pilot study. Regarding policy, we strive to offer more parents the possibility to attend the psychoeducation meeting on parenting and a mental illness at the UCP, for example by sharing our knowledge with other health care providers and governmental organizations.

In conclusion, our findings indicate that our preventive family-focused intervention met the needs of parents that participated in the psychoeducation meetings. Feedback of participants showed that they regarded the evening as useful, and more importantly, they seemed to be more aware of the potential impact of their illness on their children. However, a large number of parents were still not reached by our intervention and further study with follow-up activities are needed to measure the effects of psychoeducation on, for example, parenting, the quality of the parent-child interaction and child wellbeing. Lastly, we regard the structural embedment of interventions and continued positive reinforcement as essential elements for long-lasting attention for the prevention of mental health problems among children of parents that are treated for mental health disorders.

## Author Contributions

MP, LD, and PH set up the project, conducted the pilot study, and wrote the manuscript. CH supervised methodology, RS supervised it all.

### Conflict of Interest Statement

The authors declare that the research was conducted in the absence of any commercial or financial relationships that could be construed as a potential conflict of interest.
