# Supplementary material for: “…and How Are the Kids?” Psychoeducation for Adult Patients With Depressive and/or Anxiety Disorders: A Pilot Study
Source: Front Psychiatry. 2019 Feb 5;10:4. doi: 10.3389/fpsyt.2019.00004 (PMC6371785; doi:10.3389/fpsyt.2019.00004)
Supplement: Supplementary file 1 [file Data_Sheet_1.doc]

**EVALUATION FORM**

**PSYCHOEDUCATION ‘PARENTING AND A MENTAL ILLNESS’**

We strive to match the content of these information meetings as good as possible with the wishes and needs of the parents who are currently in treatment. Therefore, we would appreciate your feedback by filling out the questions below.

1. **How did you know about these information evenings?** (more than one answer possible)

O via my therapist

O via the website [https://kopp.umcg.nl](https://kopp.umcg.nl/)

O via de information letter or flyer on ‘Parenting and a mental illness’

O other, namely:

……………………………………....................................................

1. **Did you perceive this evening as useful?**

O yes

O no

Can you describe why you did (not) find it useful?

……………………………………....................................................

1. **After following this information evening, what are your needs regarding parenting and wellbeing of the children?** (more than one answer possible)

O this information was sufficient

O I would like to have more (practical) information, such as where I can have support in case of questions on parenting

O I would like to talk about parenting with a nurse more often

O I would like to have further conversations with a family therapist or social worker, to involve my whole family

O other, namely:

……………………………………....................................................

**This form was filled out by (name):**

**………………………………………………..**

**May we contact you for an interview about attention for parenting and a mental illness during treatment?**

- Your email address: ………………………………………………………..............
- Phone number: ……………………………………………………….........................
