# Supplementary material for: “…and How Are the Kids?” Psychoeducation for Adult Patients With Depressive and/or Anxiety Disorders: A Pilot Study
Source: Front Psychiatry. 2019 Feb 5;10:4. doi: 10.3389/fpsyt.2019.00004 (PMC6371785; doi:10.3389/fpsyt.2019.00004)
Supplement: Supplementary file 2 [file Data_Sheet_2.docx]

**Questionnaire on Parenting and a Mental Illness**

(text of the online questionnaire, translated in English)

**1. Are you a parent/caregiver of a child under the age of 24 years?**

No 🡪 you can stop filling out this questionnaire

Yes 🡪 go to question 2

**2. Who fills out this questionnaire?**

Father

Mother

Other caregiver

**3. For which mental illness are you currently following treatment?**

Anxiety disorder

Depressive disorder

Bipolar disorder

Psychotic disorder

Personality disorder

I don’t know

Other, namely:

**4. When did you start treatment for the mental illness mentioned in question 3?**

(year)

**5. On what age did you perceive mental problems for the first time?**

Before 20 years of age

Between 20 and 30 years of age

Between 30 and 40 years of age

After 40 years of age

**6. What is your family situation?**

Single

Having a partner but not living together

Living together with partner

Other, namely:

**7. How many children do you have?**

(child – sex – year of birth)

**8. Where do your children live? (more than one answer possible)**

Only at my place

They all live in the same home

Partly at my place and partly at my ex-partner’s place

My children have left home and live independently

Other, namely:

FOLLOW-UP QUESTION (only if partner ‘yes’ on question 6)

**Does your partner join you to your mental health care appointments?**

Yes, (almost) every time

At least half of the times

Less than half of the times

No

FOLLOW-UP QUESTION (only following last two options above)

**What is the reason that your partner does not join you (often)? (more than one answer possible)**

I don’t want to bother my partner with my illness

I regard my appointments as private, something for myself only

I find it difficult to talk with my partner about my illness

My partner does not have time to join me

Other, namely:

**9. Do your children know that you have mental health problems?**

Yes

I guess my children notice that there is something

I don’t think that my children suspect anything

I don’t know

No

FOLLOW-UP QUESTION (following last three options above):

**What is the reason that your children don’t know?** (more than one answer possible)

My children are too young to understand

I don’t want to bother my children

I find it difficult to talk with my children about my mental health problems

Other, namely:

**10. Do you sometimes worry about the wellbeing and/or development of your children?**

Yes

No

FOLLOW-UP QUESTION (if ‘yes’ on question 10):

**Which factors play a role in your worries?** (more than one answer possible)

That I cannot give my children the care that they need

That my children tend to care for me, instead of me caring for them

That my mental illness may be heritable and that my children will get it too

I already see that my children are developing mental health problems too

All pressure is one the shoulders of me and my partner. No one else is available to help us.

Other, namely:

FOLLOW-UP QUESTION (if ‘no’ on question 10)

**Which of the following factors ease you that your children are doing well?** (more than one answer possible)

I know where to attend on in case my children are not feeling well

If I don’t feel well my children can lean on my partner as too

In case it would be needed my children can have support of others (for example by friends or grandparents).

Other, namely:

**11. Is there attention for your role as parent/caregiver in you treatment?**

Yes

No

**12. What would you wish regarding attention for parenting in your treatment?** (more than one answer possible)

For me, it is sufficient how it is

To know how I can be supported in my parenthood

To know how I can limit the impact of my mental illness on my children’s wellbeing

To know how I can keep attention for the children regardless of my illness

To know how I can talk with my children about my mental illness

Other, namely:

**13. Would you like to have further information about options for family support? Your therapist or the social worker could tell you more about it.**

Yes

No

**14. Do you want that your children receive more information about the mental illness, for example in an individual meeting, an online knowledge and chat platform, or a course in a peer group?**

Yes

No

**15. Did you attend the psychoeducation meeting ‘Parenting and a Mental Illness’? This meeting is given every month.**

Yes

No

FOLLOW-UP QUESTION if ‘yes’: What brought you to this psychoeducation meeting? [open question]

FOLLOW-UP QUESTION if ‘no’: What is the reason that you did not go to the meeting? [open question]
