# Supplementary material for: “…and How Are the Kids?” Psychoeducation for Adult Patients With Depressive and/or Anxiety Disorders: A Pilot Study
Source: Front Psychiatry. 2019 Feb 5;10:4. doi: 10.3389/fpsyt.2019.00004 (PMC6371785; doi:10.3389/fpsyt.2019.00004)
Supplement: Supplementary file 3 [file Data_Sheet_3.docx]

**Online survey ‘KOPP’ (‘COPMI’), translated in English**

All questions were multiple choice with the possibility to explain the answer in a text box.

Question 1.

What is your current function in the University Center Psychiatry?

Question 2.

On which outpatient and/or inpatient ward are you working?

Question 3.

If it is known that a patient has children between 0 and 24 years of age, do you bring up the theme parenting in conversations with the patient?

Question 4.

With how many of your last ten patients with children under the age of 24 have you (ever) talked about parenting during their treatment?

Question 5.

Looking at the same last patients that you have seen, how many parents have you informed about the psychoeducation for parents, which is organized by the University Center Psychiatry in collaboration with Accare (child en youth psychiatry)?

Question 6.

Which moment in the timeline of the treatment do you find most appropriate for bringing up the theme parenting?

Question 7.

Do you experience barriers in talking about parenting, with regard to your current function?

Question 8.

Do you have the need for more knowledge about and/or skills training in talking about parenting, with regard to your current function?

Question 9.

Are you familiar with the guideline ‘Children of parents with mental illnesses’ (National Trimbos Institute)?

Question 10.

Imagine that you are having a conversation with one of your parent-patients. In this conversation, you notice signals of a threatened developmental situation for the child. Would you know what to do?
